# The Effect of Cone Localization on Higher Order Aberrations After Corneal Crosslinking for Keratoconus

**DOI:** 10.14744/bej.2021.07088

**Published:** 2021-09-27

**Authors:** Nilay Kandemir Besek, Gulay Yalcinkaya, Ahmet Kirgiz, Fevziye Ondes Yilmaz, Burcin Kepez Yildiz, Yusuf Yildirim, Ahmet Demirok

**Affiliations:** Department of Ophthalmology, University of Health Sciences, Beyoglu Eye Training and Research Hospital, Istanbul, Turkey

**Keywords:** Central cone, cone localization, crosslinking, high order aberrations, keratoconus

## Abstract

**Objectives:**

This study was designed to evaluate the effects of corneal collagen cross-linking (CXL) on topographic parameters, visual acuity, and corneal high-order aberrations according to the preoperative cone location in keratoconus.

**Methods:**

This retrospective study assessed patients with keratoconus who underwent CXL between March 2016 and February 2019. Patients with a history of corneal surgery, corneal hydrops, corneal scar tissue, delayed epithelial healing, and a corneal thickness of <400 μm were excluded. The included eyes were divided into 2 groups according to the preoperative cone location: maximum K in the central 3-mm optical zone (group 1) or the central 3-mm to 5-mm optical zone (group 2). The preoperative and postoperative 24-month, best-corrected visual acuity (BCVA), intraocular pressure, K max, symmetry index front, corneal thickness, and high order aberration findings were recorded.

**Results:**

The study included 67 eyes of 67 patients with keratoconus: 39 in group 1, and 28 in group 2. There were statistically significant differences between the groups in the preoperative BCVA values (p=0.04). There was no significant difference between the preoperative and postoperative mean K max between the 2 groups (p=0.08). The mean difference in corneal thickness between preoperative and postoperative measurements was significantly lower in group 2 than in group 1 (p=0.03). The preoperative and postoperative mean spherical aberration was significantly higher in group 1 than in group 2 (p=0.001 and p=0.005, respectively).

**Conclusion:**

The preoperative cone location in keratoconus may affect CXL outcomes. At the end of the second year, CXL was found to have a similar efficacy on visual acuity and keratometry parameters in the central and paracentral cone groups, and the recovery in terms of a spherical aberration among high-order aberrations after CXL in the central cone group was better than that of the paracentral cone group.

## Introduction

Keratoconus is progressive, bilateral, and asymmetrical ectasia of the cornea (1, 2). Decreased visual acuity is directly related to irregular astigmatism and dense corneal opacities. When optical devices such as glasses or hard lenses no longer provide comfort and acceptable visual quality, surgical treatments such as intracorneal rings or corneal transplants may be needed. Corneal collagen cross-linking (CXL) is a minimally invasive method that delays the need for surgery by slowing the progression of keratoconus. Cross-linking of corneal collagen is among medical treatments and more aggressive surgical treatments (3). A photodynamic reaction caused by ultraviolet A (UVA) light and riboflavin making it photosensitive causes an enhancement in the number of bonds and collagen resistance to enzymatic breakdown (4, 5).

Although there are several studies on the effectiveness of CXL treatment in the literature, studies evaluating its effect on different keratoconus topography patterns are limited. In the literature, the impact of properties of corneal topography on the outcomes of CXL has not been presented. Due to the influence of CXL could be biomechanical, the original topography could be significant to the effect of CXL. The effect of CXL on corneal biomechanics at different cone locations may be significant and topographic cone localization in keratoconus may affect CXL treatment results. In this study, we evaluated the possible influence of CXL on topographic parameters, visual acuity, and corneal high-order aberrations according to pre-operative cone location.

## Methods

The consecutive patients with keratoconus who were treated with CXL between February 2016 and January 2019 in the University of Health Sciences Turkey, Beyoglu Eye Training and Research Hospital, Istanbul, Turkey, were analyzed in this retrospective study. Approval was gotten from the Okmeydani Training and Research Hospital Ethics Committee for the study, and written informed consent was received from all patients included in the study in accordance with the principles of the Declaration of Helsinki.

Patients who aged 14 years or older and axial topography consistent with keratoconus were included in the study. If there were one or more of the following changes 12 months, it was defined as progressive keratoconus: An increase of ≥1.00 D in the manifest cylinder, an increase of ≥1.00 D in the steepest keratometry, or an increase of ≥0.50 D in manifest refraction spherical equivalent. Patients with a history of ocular surgery and previous ocular history, such as corneal hydrops, corneal scar tissue, conjunctivochalasis, delayed epithelial healing, chemical or penetrating injury, pregnancy and breastfeeding, and corneal thickness <400 μm, were excluded from the study.

All eyes were treated with accelerated CXL procedure. After the topical administration of 0.5% proparacaine hydrochloride (Alcaine; Alcon Laboratories, Inc., Fort Worth, TX, USA), using a blunt spatula, the central epithelium was peeled. After epithelial removal, riboflavin (0.1% solution VibeX; Avedro Inc., Waltham, MA) was dropped every 2 min for a total of 20 min. The corneal stroma was exposed to 365 nm UVA light (Peschke; Meditrade CCL-Vario system) over a 9 mm diameter and with an irradiance of 9 mW/cm^2^ for 10 min, following riboflavin installation. Then, the ocular surface was washed with 20 mL of a balanced salt solution, medicated with 0.5% moxifloxacin (Vigamox; Alcon Laboratories, Inc., Fort Worth, TX, USA.) and a silicone hydrogel bandage contact lens (Air Optix; Alcon Laboratories, Inc., Fort Worth, TX) was applied until the closure of the epithelial defect. Postoperatively, 0.5% moxifloxacin (Vigamox; Alcon Laboratories, Inc., Fort Worth, TX, USA.) was given 5 times a day for 5 days, artificial tears without preservatives were given 5 times a day for 4 weeks, and 0.5% loteprednol etabonate (Lotemax; Bausch and Lomb, USA) was applied 4 times a day and tapered over 1 month.

Topography measurements were obtained using the Sirius (Costruzione Strumenti Oftalmici, Firenze, Italy). According to the location of cone preoperatively, defined by the Sirius coordinates of maximum K, eyes were separated into two groups: Those eyes in which the maximum K was located in the central 3 mm optical zone (Group 1: central cone) and central 3 mm–5 mm optical zone (Group 2: paracentral cone). Data of maximum K were gotten preoperatively and at 24 months postoperatively. Pre-operative and post-operative 24 months uncorrected and best-corrected visual acuities (and BCVA, respectively), intraocular pressure, posterior segment findings, K max, symmetry index front (zero), corneal thickness, and high-order aberrations (HOA) were noted.

### Statistical Analysis

Statistical analysis was performed by SPSS (version 22.0, SPSS, Inc., Chicago, IL, USA). All data were normally distributed according to the Kolmogorov–Smirnov test. The Chi-square test was used for categorical variables. The Student’s t-test independent was used for independent variables and Student’s t paired test was used to compare pre-operative and post-operative data. Before statistical analysis, visual acuity was converted to the logarithm of the minimum angle of resolution. P<0.05 was considered statistically significant.

## Results

In the study, there were 67 eyes of 67 patients with keratoconus (37 males and 30 females), of which 39 eyes of 39 patients (18 males and 21 females) with central cone were classified as Group 1, and 28 eyes of 28 patients (19 males and 9 females) with the paracentral zone were categorized as Group 2. Between the two groups, there was no significant difference in terms of sex (p=0.08). The mean age was 24.4±4.24 years (range: 15–35 years). The mean ages were 23.62±0.76 in Group 1 and 25.5±0.59 years in Group 2 (p=0.072). Comparison of visual acuities and corneal topographic findings between the two groups is shown in Table 1. Although there were statistically significant differences between the two groups in terms of pre-operative BCVA values (p=0.04), there were no statistically significant differences in terms of post-operative BCVA values (p=0.37). Besides, there was a statistically significant increase in BCVA postoperatively compared to pre-operative ones in Group 1 (p=0.02).

**Table 1.** Comparison of visual acuities and corneal topographic findings between the two

		**Group 1 (n=39)**	**Group 2 (n=28)**	**p**
UCVA			
Preop	0.66±0.54	0.57±0.8	0.37
24 month postop	0.64±0.56	0.61±0.8	0.8
Mean change	0.2±0.31	-0.4±0.41	0.23
P^*^	0.5	0.34	
BCVA (LogMAR)			
Preop	0.43±0.51	0.28±0.48	0.04
24 month postop	0.34±0.5	0.27±0.55	0.37
Mean change	0.09±0.39	0.01±0.39	0.14
P^*^	0.02	0.77		
Kmax (D)			
Preop	56.44±0.8	54.42±0.78	0.08
24 month postop	55.58±0.78	53.62±0.69	0.08
Mean change	0.87±0.18	0.8±0.37	0.86
P*	0.001	0.04	
SIf (D)			
Preop	5.31±0.48	5.98±0.46	0.33
24 month postop	4.91±0.43	5.53±0.41	0.32
Mean change	0.4±0.18	0.46±0.19	0.8
P*	0.03	0.02	
Thin (mμ)			
Preop	459.33±7.62	463.43±6.15	0.69
24 month postop	439.64±7.85	453.36±5.52	0.19
Mean change	19.69±3.43	10.07±1.98	0.03
P*	0.001	0.001	

UCVA: Uncorrected visual acuity; BCVA: Best-corrected visual acuity; Kmax: Maximum keratometry; SIf: Symetry Index front; D: Diopters, Thin: Thinnest point of cornea. P: Student’s t-test independent; P*: Student’s t-paired test; Values in bold are significant (p<0.05).

There was no statistically significant difference between the pre-operative and post-operative mean K max between the two groups (p=0.08). The mean pre-operative K max was 56.44±0.80 D in Group 1. At the 1^st^ year, K max was significantly reduced by 0.87 D to 55.58±0.78 D (p=0.001). In Group 2, the mean pre-operative K max was 54.42±0.78 D. At the 1^st^ year, K max was significantly decreased by 0.80 D–53.62±0.69 D (p=0.004). The difference between pre-operative and post-operative corneal thickness was not found statistically significant in Group 1 and Group 2 (p=0.69 and p=0.19, respectively). The mean difference of corneal thickness between pre-operative and post-operative was significantly lower in Group 2 than Group 1 (10.07±1.98 and 19.69±3.43, respectively, p=0.03).

In Group 1 and Group 2, a significant decrease was observed in total, trefoil, coma, and spherical aberrations compared to pre-operative values at the 24^th^ month post-operative (p=0.001). The decrease in spherical aberrations was found significantly more in Group 1 compared to Group 2 (p=0.004) (Table 2).

**Table 2.** Preoperative and 24-month postoperative corneal high order aberrations

**HOAs (6-mm)**	Group 1 (n=39)	Group 2 (n=28)	p
Total (RMS, μm)			
Preop	1.81±0.13	1.42±0.12	0.04
24 month postop	0.91±0.67	0.7±0.57	0.03
Mean change	0.9±0.11	0.71±0.09	0.24
P*	0.001	0.001	
Trefoil (RMS, μm)			
Preop	0.61±0.05	0.48±0.51	0.08
24 month postop	0.39±0.04	0.29±0.27	0.04
Mean change	0.22±0.05	0.19±0.37	0.68
P*	0.001	0.001	
Coma (RMS, μm)			
Preop	1.5±0.12	1.26±0.11	0.16
24 month postop	0.71±0.06	0.58±0.57	0.13
Mean change	0.79±0.1	0.68±0.08	0.42
P*	0.001	0.001	
Sferik (RMS, μm)			
Preop	0.41±0.07	0.13±0.01	0.001
24 month postop	0.14±0.02	0.07±0.01	0.005
Mean change	0.27±0.59	0.06±0.01	0.004
P*	0.001	0.001	

HOAs: Higher order aberrations; RMS: Root mean square. P: Student’s t-test independent; P*: Student’s t-paired test; Values in bold are significant (p<0.05).

## Discussion

CXL treatment, which has been used for many years to prevent the progression of keratoconus, is a proven method (6-8). In recent years, accelerated CXL treatment protocols have come to the fore. However, both treatment protocols are not applied according to the degree of keratoconus or the cone location. There are a limited number of publications in the literature about the effectiveness of CXL in different cone localization. In the current study, we evaluated the effect of CXL on the central and paracentral cones. At the end of 24 months, we found that CXL had similar efficacy on visual acuity and keratometry parameters in the central and paracentral cone groups, and, the recovery in terms of a spherical aberration among high-order aberrations after CXL in the central cone group was better than the paracentral cone group.

K max value has been reported as a substantial parameter in evaluating the effectiveness of CXL (9). Raiskup-Wolf et al., (10) in a study they conducted in 2008, found a decrease of 2.68 D in K max in 1 year, Hersh et al. (11) found a 2 D decrease in K max in their prospective studies. Steven et al. (12) reported that preoperatively, a higher K max was associated with a probability of more potent corneal flattening through CXL. They found that eyes with a pre-operative K max ≥55.00 D were 2.7 times more likely to flatten by ≥2.00 D, 1 year after CXL. They assumed that their results could be related to the central cones being steeper than the paracentral cones. They also reported that there was a 2 D decrease in K max in the central zone and a 0.4 D decrease in the peripheral zone (12). In the same study, the difference of CXL in topographic results according to cone localization was tried to be explained by some mechanisms. When the process is conducted using the UV technique, the entire area may not be evenly treated (13). The peripheral cornea may not be exposed to the same UV energy profile. Even if the homogeneity would not be an issue, the “cosine effect” might cause the peripheral cornea to be less treated with CXL. Since the angle of the incidence of the beam with the cornea is reduced peripherally, the beam energy is distributed to a larger corneal surface, due to corneal curvature. Therefore, it results in reduced treatment per unit are and thus less cross-linking response in peripheral cones (14). Although we found a significant decrease in K max level in the central and paracentral cone groups in our study (0.87D and 0.80 D, respectively), there was not a significant difference between the Group 1 and Group 2.

Although CXL treatment does not aim to improve visual acuity, changes in corneal topography may cause this improvement secondary to that. O’Brart et al. (15) reported that CXL continues to support the efficacy of this treatment in progressive keratoconus, with an improvement in BCVA 12 months after CXL. Although the relationship between cone localization and BCVA has not been fully demonstrated, Steven et al. (12) reported that patients with central cone localization had lower initial visual acuity and higher K max, and post-operative BCVA might be lower accordingly. In this way, as compared to peripheral cones, even a significant recovery in keratometry may not affect BCVA widely enough to represent a statistical difference from the others (12). In our study, pre-operative visual acuity was lower in Group 1, and when the two groups were compared, the increase in BCVA in Group 1 was found to be higher than in the group.

Corneal aberration values decrease after CXL (16-18). HOAs may be expected to prognosticate recovery in vision after CXL. Increased HOA values as a result of keratoconus cause a decrease in visual acuity. Contrarily, correcting HOAs by CXL in keratoconus can increase visual acuity (19). El-Massry et al. (20) were described HOAs for a 6 mm diameter. The total HOAs root mean square was 2.05±1.55 μm preoperatively. It had significantly reduced to 1.36±1.25 μm at 6 months. In the same study, a statistically significant difference was reported for coma and spherical aberration in the post-operative 6^th^ month (20). It is unclear that these findings in their study show whether ectatic corneas improve less with CXL than corneas in keratoconus and whether pre-operative topographic data play a role in this difference. In our study, in accordance with the literature, a decrease in total trefoil, coma, and spherical aberrations was observed in both groups. However, improvement in spherical aberrations was higher in Group 1 than in Group 2. The higher improvement in spherical aberration in the central cone group compared to the paracentral group may have been due to the greater effect of CXL treatment on the central cone. In our study, we detected that corneal HOAs, in particular spherical, improved after corneal CXL in both groups.

Although our study contributed to the literature by evaluating the changes in HOA after CXL treatment in keratoconus according to the location of the cone, it has some limitations: It is retrospective, the number of patients is relatively low, and there is no peripheral cone group.

As a result, we have shown that CXL treatment provides greater improvement in spherical aberration values in cases with topographic central cone localization in keratoconus. Further prospective studies are necessary to define whether the pre-operative location of the cone influences other CXL effects and whether pre-operative cone location should be more significant in CXL therapy for keratoconus.

## Disclosures

### Ethics Committee Approval:

Istanbul Prof. Dr. Cemil Tascioglu City Hospital Ethics Committee, protocol number: 326, Date: 14/07/2020.

### Peer-review:

Externally peer-reviewed.

### Conflict of Interest:

None declared.

### Authorship Contributions:

Involved in design and conduct of the study (NKB, AK, BKY); preparation and review of the study (NKB, GY, AD); data collection (GY, FOY, YY); and statistical analysis (NKB, AK, YY).
